# Liquid Biopsy as a Source of Nucleic Acid Biomarkers in the Diagnosis and Management of Lynch Syndrome

**DOI:** 10.3390/ijms23084284

**Published:** 2022-04-13

**Authors:** Gergely Buglyó, Jakub Styk, Ondrej Pös, Ádám Csók, Vanda Repiska, Beáta Soltész, Tomas Szemes, Bálint Nagy

**Affiliations:** 1 Department of Human Genetics, Faculty of Medicine, University of Debrecen, 4032 Debrecen, Hungary; csok.adam@med.unideb.hu (Á.C.); soltesz.beata@med.unideb.hu (B.S.); nagy.balint@med.unideb.hu (B.N.); 2 Institute of Medical Biology, Genetics and Clinical Genetics, Faculty of Medicine, Comenius University, 811 08 Bratislava, Slovakia; jakub.styk@gmail.com (J.S.); vanda.repiska@fmed.uniba.sk (V.R.); 3 Comenius University Science Park, Comenius University, 841 04 Bratislava, Slovakia; ondrejpos.sk@gmail.com (O.P.); tomasszemes@gmail.com (T.S.); 4 Geneton Ltd., 841 04 Bratislava, Slovakia; 5 Department of Molecular Biology, Faculty of Natural Sciences, Comenius University, 842 15 Bratislava, Slovakia

**Keywords:** lynch syndrome, colorectal cancer, screening, liquid biopsy, circulating nucleic acids, biomarker

## Abstract

Lynch syndrome (LS) is an autosomal dominant inherited cancer predisposition disorder, which may manifest as colorectal cancer (CRC), endometrial cancer (EC) or other malignancies of the gastrointestinal and genitourinary tract as well as the skin and brain. Its genetic cause is a defect in one of the four key DNA mismatch repair (MMR) loci. Testing of patients at risk is currently based on the absence of MMR protein staining and detection of mutations in cancer tissue and the germline, microsatellite instability (MSI) and the hypermethylated state of the MLH1 promoter. If LS is shown to have caused CRC, lifetime follow-up with regular screening (most importantly, colonoscopy) is required. In recent years, DNA and RNA markers extracted from liquid biopsies have found some use in the clinical diagnosis of LS. They have the potential to greatly enhance the efficiency of the follow-up process by making it minimally invasive, reproducible, and time effective. Here, we review markers reported in the literature and their current clinical applications, and we comment on possible future directions.

## 1. Introduction

Lynch syndrome (LS), first described by Henry T. Lynch in 1966 [1], also known as a hereditary non-polyposis colorectal cancer syndrome (HNPCC), is one of the most prevalent cancer-prone syndromes associated with a high probability of developing multiple synchronous and/or metachronous malignancies with relatively early onset [2]. The term HNPCC is recently used less often because individuals with LS may develop precancerous colorectal polyps [3,4]. Typically, these polyps appear in early adulthood and may eventually undergo malignant transformation [5]. LS may manifest as colorectal cancers (CRCs) only, or increase the risk of extracolonic malignancies, such as endometrial, ovarian, stomach, urinary tract, small intestine, pancreas, and hepatobiliary tract neoplasms.

The condition is inherited in an autosomal dominant pattern, showing a high penetrance and variable expressivity [6,7]. Among all CRC cases, approximately 3–5% are attributed to this syndrome [8]. It is caused by a heterozygous germline mutation at one of the loci regulating post-replicative DNA mismatch repair (MMR), such as mutL homologue (MLH1), mutS homologue 2 (MSH2), mutS homologue 6 (MSH6), or postmeiotic segregation increased 2 (PMS2) [9]. Deletions involving the last exons of the epithelial cell adhesion molecule gene (EPCAM) were also reported [10]. Alterations at the 3′ end of EPCAM may produce a read-through EPCAM/MSH2 fusion transcript while silencing the native MSH2 promoter by hypermethylation [11,12]. Mutations of MSH2 (or rarely MLH1, MSH6 or PMS2) may cause a subtype known as Muir–Torre syndrome, featuring sebaceous adenomas and keratoacanthomas of the skin [13,14,15,16,17,18]. In a subset of carcinomas, MMR deficiency (MMR-D) is found, but no germline mutation can be detected in the underlying MMR gene. This group has been called ‘Lynch-like’ [19].

To increase the efficiency of screening and follow-up, insight is needed on cancer risk and survival at different ages of mutation carriers for all four MMR genes [2,20]. Prospective Lynch Syndrome Database (PLSD) provides such data, showing a detailed cumulative risk of CRC, endometrial carcinoma (EC) and ovarian cancer at 70 years in both sexes [21]. The incidence of malignancies is reported to be much lower in PMS2 mutation carriers (cumulative lifetime risk has been estimated at 18.75% in males and 10.56% in females for CRC and 11.78% for EC at age 70 [22]) compared to the other MMR genes [20].

The worldwide prevalence of LS is estimated at around 0.35%. In Western populations, the frequency of LS patients is reported between 1:370 and 1:2000 [23]; however, LS may be even more prevalent as suggested by Cerretelli et al. (1:100–1:180) [24]. Nearly all (95%) affected individuals are unaware of their disease and are at considerable risk of developing CRC or other malignancies [25], depending on the mutated MMR gene [26,27]. Loss of PMS2 function has the highest prevalence at 0.140%, which is followed by MSH6 at 0.132%, while MLH1 and MSH2 mutations are the rarest at 0.051% and 0.035%, respectively [28]. There is considerable variation in prevalence data among human populations due to different founder mutations [29].

In this paper, we review the long-persisting challenges and latest advancements in the clinical management of LS. Due to a lack of distinct symptoms during the early phases of malignant disease, developing a reliable and minimally invasive screening method is essential. We discuss proposed cell-free DNA-based and RNA-based biomarkers obtainable from liquid biopsies and their possible roles in the diagnosis of LS, offering some predictions on future developments in the field.

## 2. Current Challenges of Lynch Syndrome Diagnosis and Follow-Up

According to the clinical criteria, the detection of LS is necessary in two situations. Firstly, after the surgical procedure, the samples are studied to see whether a tumour is sporadic or produced by inherited MMR deficiency [30], which is associated with microsatellite instability (MSI) [31]. Secondly, direct germline testing is performed on individuals whose family history indicates the possibility of LS [30]. Today, next-generation sequencing (NGS) is much faster and more cost-efficient than traditional sequencing [32]. Growing evidence supports the view that tumour sequencing, as the first step in LS and polyposis syndrome screening, could easily replace conventional testing of patients with CRC [33].

Testing CRC and EC cases is routinely performed to reveal if LS is in the background. Apart from mutations found in MMR genes, MSI is seen as a tumour-specific marker for LS when observed in malignant tissue and less clearly so in adenomas [34]. MSI refers to changes in the length of repetitive DNA sequences called short tandem repeats (STRs) or microsatellites in tumour samples compared with normal non-neoplastic tissue [35]. In current clinical practice, five quasi-monomorphic mononucleotide markers (BAT-25, BAT-26, NR-21, NR-24 and MONO-27) are routinely analysed by gold standard MSI-PCR fragment length analysis (Promega^®^ MSI Analysis System), and in case more than 20% are found to be unstable (or ≤2 markers), the tumour is classified as MSI-high (MSI-H). In MSI-low (MSI-L) tumours, only one locus shows instability, while if the fragmentation profiles match at all tested loci in paired tissue samples, the tumour is considered microsatellite stable (MSS) [36]. Apart from being a sign for LS, MSI-H is also a marker showing the tumour’s responsiveness to immunotherapy [35]. Studies have found that colorectal malignancies with a diagnosed MSI-H status, aberrant in the function of the MMR system, respond better to personalised immune therapy than tumours with a low degree of instability. This finding applies to both sporadic and hereditary forms of CRC [37].

NGS platforms are suitable to detect MSI status. Recently, there have been attempts to use NGS technology to assess more microsatellite loci than conventional gold standard methods [38]. As recently reviewed by Gilson et al. (2021), various methods have been tailored for MSI testing and completed with the possibility of detection of hotspot mutations in the KRAS, NRAS and BRAF genes [39]. All sequencing approaches, including whole-genome, whole-exome, targeted-genome and RNA sequencing, are valid for MSI testing [40]. However, analysing each locus separately may not provide sufficient information on MSI events at the whole-genome level [39]. In an era when whole-genome sequencing tests are already used in clinical practice (e.g., in non-invasive prenatal testing) [41], the idea of global screening for MSI events is becoming increasingly realistic.

Although the MSI-H phenotype is well characterised in CRC and EC, it has been observed in a broad spectrum of other tumour types, and the prevalence of MSI events varies significantly with recent studies observing cancer-specific MSI patterns [42,43,44]. There are a few web-based tools available for microsatellite identification, e.g., MISA predictor (MIcroSAtellite identification tool) and its improved web-based application [45], GMATo (Genome-wide Microsatellite Analysing Tool) [46] and PolyMorphPredict [47].

As a pre-screening procedure for LS and MMR status evaluation, four proteins are usually detected by immunohistochemical staining (IHC): MLH1, MSH2, MSH6 and PMS2. An absence of staining shows a dysfunction of the protein or its production [48]. When the MLH1 protein is not visible after labelling by a monoclonal antibody, MLH1 promoter analysis is the next step [49]. Methylation of this promoter is common in elderly patients and accounts for about 70% of MLH1- and PMS2-negative CRC [50] and 94% of EC cases [51]. MLH1 promoter methylation status is usually assessed before germline mutation testing. Reliable detection of MSI status is necessary to select CRC patients who may benefit from immunotherapy such as PD-1/PD-L1 (programmed cell death 1/programmed cell death ligand 1) blockade therapy [52].

In contrast to MSI-L and MSS, MSI-H tumours are characterised by the highly upregulated expression of various immune system checkpoints [53,54]. In metastatic CRC (mCRC), approximately 10% of patients have been shown to be incorrectly included in immunotherapeutic studies due to false positive IHC or MSI-PCR results [55]. Therefore, we prepared an outline of the major advantages and limitations of the aforementioned methods to highlight the possibility of mismanagement of LS-suspected patients using traditional tissue-based approaches (Table 1). The presence of non-truncating and/or truncating pathological mutations in MMR genes may lead to false negative results (staining present due to antigenicity being intact while the protein’s actual function is disrupted), so an additional PCR analysis is required for the correct interpretation of patients’ MSI status [56].

Germline testing of MMR genes is performed even in the case of MMR-D tumours, as 70% of them are caused by biallelic somatic inactivation and/or epigenetic silencing [57]. To confirm the diagnosis of LS, testing for the somatic BRAF V600E mutation is useful, as it is found in 69–78% of CRC patients with MLH1 promoter methylation [58] and not present in the vast majority of LS cases [59]. It has been suggested that with the combined analysis of the MLH1 promoter and BRAF, the number of cases in which germline MMR gene testing is inevitable could be reduced by half [60].

Currently, pedigree criteria and DNA sequencing are standard methods to identify LS patients. Interestingly, some families that (based on clinicopathological features) meet the Amsterdam I/II [61,62] or revised Bethesda criteria [36] for LS screening do not show a mutation in any of the known MMR genes, while some individuals have been shown to harbour MMR defects despite not fulfilling the criteria [63]. MSH6 mutation carriers do not necessarily satisfy these screening criteria, as they tend to develop CRC at an older age than MLH1 or MSH2 mutation carriers and have reduced penetrance [64,65,66]. These discrepancies lead to a need to improve clinical guidelines. The Jerusalem criteria, a new guideline established in 2009, differs from widely used criteria in that it recommends any CRC case for MSI testing if the patient is <70 years old [67]. One of the latest versions of the screening criteria, presented by the National Comprehensive Cancer Network (NCCN), recommends universal screening for all patients with CRC and EC showing signs of MMR-D at any age of diagnosis [68]. In cases or families where no tumour sample is available but clinical signs are present, predictor algorithms such as PREMM, MMRpro, and MMRpredict may prove helpful [69,70,71].

If molecular methods confirm LS, there are two possible strategies for CRC screening in current clinical practice. Despite steady progress in innovative and less invasive approaches, colonoscopy is still considered a gold standard of colorectal surveillance in LS screening [72]. The procedure is generally considered safe, but rare complications are poorly presented. Possible risks were reported in a nested case-control study covering nearly 40,000 colonoscopies [73]. Colonoscopy performed more than once every three years did not reduce colorectal cancer incidence or stage at diagnosis nor did it enhance survival [20]. The lack of specific quality standards for colonoscopy screening is responsible for the high number of missed cases [74].

Faecal occult blood tests (gFOBT: guaiac-based faecal occult blood test; FIT or iFOBT: immunochemical faecal occult blood test and faecal DNA test) are an alternative to be considered if colonoscopy is rejected. The limited sensitivity of FIT tests complicates the detection of bleeding, and some studies have reported increased false-negative FIT results in participants with a family history of CRC [75,76,77].

To sum it all up, LS is currently characterised by a somewhat elusive diagnosis and invasive procedures during follow-up that often result in poor patient compliance. There is a growing need for simple non-invasive sampling such as liquid biopsy, allowing to obtain tumour cells or cell-free nucleic acids (cfNAs). cfNAs extracted from liquid biopsies: (i) may have a limited application in the diagnosis of LS; and (ii) may greatly improve the follow-up process by non-invasive testing, which may be performed more frequently.

## 3. Liquid Biopsy as a Source of Cell-Free Nucleic Acids

The current trend in oncology is moving towards minimally invasive approaches for the early diagnosis, ongoing monitoring and prediction of the therapeutic response in cancer patients [78]. Tumour biopsy as the gold standard for the histological analysis, mutation and MSI analysis in sporadic and LS-associated CRCs is not without limitations. Location of a tumour sample may (i) make it difficult to access; (ii) a sample may not give information about the whole-genome state of the disease due to intra- and inter-tumour heterogeneity; (iii) a single tissue biopsy may result in MSI misclassification; and (iv) repeated sampling is not possible [39].

**Figure 1:**
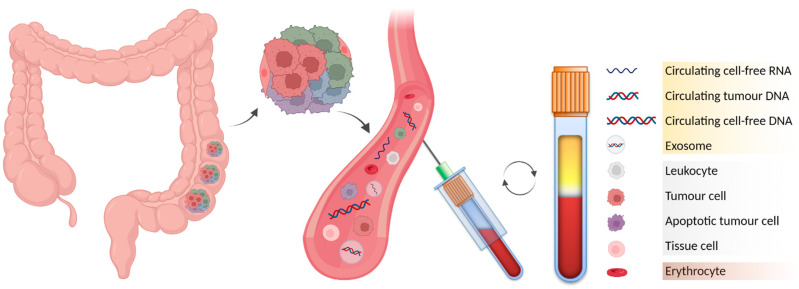
Blood-based liquid biopsy as a reservoir of circulating nucleic acid biomarkers. Analysis of peripheral blood allows the study of intra- and inter-tumour heterogeneity via analysis of cfNAs. As soon as possible after collection, whole blood should be separated by centrifugation to obtain the following fractions: (i) upper plasma layer (yellow), (ii) intermediate buffy coat (white), and (iii) bottom layer of erythrocytes (red). (Created with BioRender.com).

In LS screening and MSI evaluation, there are countless potential benefits of blood-based genomic profiling over conventional methods. Liquid biopsy is a simple, repeatable, inexpensive and relatively painless method to collect samples. It is expected to become the cornerstone of personalised treatment plans in the future, based on individual genetic variation in many types of malignancy [79]. It may be used to follow up a patient’s treatment efficacy and allow tumour detection in cases where family history increases the risk of developing cancer [80,81]. Liquid biopsy, particularly peripheral blood, contains adequate amounts of (i) circulating tumour cells (CTCs); (ii) fragments of circulating tumour DNA (ctDNA) derived from primary and/or secondary tumours; (iii) other circulating cell-free DNA (cfDNA) of nuclear and mitochondrial origin; (iv) circulating cell-free RNAs (cfRNAs) including messenger RNA (mRNA), microRNA (miRNA), long non-coding RNA (lncRNA) and circular RNA (circRNA); and (v) extracellular membrane vesicles (EMVs) such as exosomes loaded with DNA or RNA molecules (Figure 1) [80,81,82,83].

Liquid biopsy may also prove useful in dealing with tumours displaying genetic heterogeneity, affecting detection, prognosis and treatment [84]. The efficiency of evaluating multiple ctDNA fractions has already been reported for the treatment-related genetic heterogeneity of mCRC [85].

Liquid biopsy-based genotyping begins with a workflow that needs careful consideration and includes many pre-analytical steps. Protocols for collecting, storing and transporting different body fluids, sample processing, cfNA extraction and data analysis are still poorly reported. Some authors have comprehensively compared laboratory protocols, methodological and technical issues, and pre-analytical processes that may eventually influence future downstream applications and the clinical utility of cfNAs as potential biomarkers in LS follow-up [86,87]. Some relevant biomarkers are outlined in Table 2.

## 4. Cell-Free DNA

Circulating cfDNA was reported more than 70 years ago [108]. It was observed that the amount of cfDNA in the peripheral blood of cancer patients is higher (up to 180 ng/μL in advanced stages) compared to healthy individuals (13 ng/μL) [109]. At present, cfDNA obtained from liquid biopsies is suitable for detecting MMR mutations, MSI and MLH1 promoter methylation status, and universal CRC markers (in the follow-up of LS). In the context of LS screening, there is growing evidence of a high concordance between MSI phenotype in cfDNA and tumour tissue. Sensitivity for the detection of methylated DNA is high (up to 90%) but was generally considered to be lower for cfDNA mutation analysis (40–60%) just a few years ago [110]. The main reason is that liquid biopsy samples contain a low quantity of highly fragmented DNA molecules. Techniques are being developed to overcome these limitations and enrich the DNA concentration of the samples before applying PCR-based methods to improve the detection of low-frequency alleles. One of the methods made available recently to improve the detection limit is nuclease-assisted minor allele enrichment with probe overlap [111]. Another method combines inter-Alu-PCR’s advantage with targeted NGS-based molecular profiling, which is called inter-Alu-PCR-NGS [88]. Elimination of the necessity for paired-sample evaluation is a major challenge before introducing blood-based MSI testing into diagnostic laboratories. New tools such as MSIsensor-ct [89] may push the boundaries of specificity and sensitivity of cfDNA-based tests in MSI analysis to 100% with 0.05% ctDNA content. This NGS-based computational tool is compatible with various sequencing methods and custom-designed gene panels.

Drop-off ddPCR (droplet digital PCR) provides clonal amplification with absolute quantification of the required MSI sequences with 100% specificity and sensitivity, as seen in the case of the BAT26, ACVR2A and DEFB105A/B microsatellites detected from CRC tissue and liquid biopsy samples [90]. A ddPCR assay has also been developed to assess promoter methylation of the MLH1 gene with convincing efficiency, even from 1 ng of cfDNA. After optimising criteria for accepting a sample as positive, distinguishing methylated CRC and healthy donor samples was performed at 78% sensitivity and 100% specificity. Differentiating between CRC samples of different methylation levels was also possible [91]. A commercially available Bio-Rad ddPCR MSI assay (a pentaplex method for the conventionally used Bethesda/NCI panel of five markers [36]) shows a performance comparable to gold standard techniques without the need to test a healthy tissue sample [92].

Of all CRC patients, approximately 20% develop liver metastases, and up to 55% are affected by metachronous metastases [112]. Clinical outcome following the resection of colorectal liver metastases (metastasectomy) is generally poor, with an overall survival of less than five years [113,114]. Metastases are clearly the most common cause of CRC death. However, no relevant nucleic acid biomarker is used in clinical practice to identify patients that may benefit from surgery in mCRC. To date, only a few studies have aimed to assess minimal or molecular residual disease in the post-metastasectomy setting in mCRC patients by testing biomarkers obtained from liquid biopsy [115].

Blood sampling is not the only form of liquid biopsy that may produce ctDNA relevant for LS screening. Mutations in the telomerase reverse transcriptase (*TERT*) promoter and the fibroblast growth factor receptor 3 (*FGFR3*) gene are sometimes seen in LS and have been proposed as novel biomarkers of urothelial cancer (UC), which is the third most common cancer type in certain subsets of LS families [116,117]. They are ideal candidates to be studied from ctDNA extracted from urine liquid biopsies [93]. Bile is another rarely utilised source of ctDNA; mutations have been reliably detected from such samples by targeted deep sequencing [118]. As up to 4% of LS patients develop bile duct cancer, bile liquid biopsies may become useful for screening in the future as new methods are being developed for cheap, non-invasive bile capture [119].

Unlike DNA of nuclear origin, mitochondrial DNA (mtDNA) is relatively rarely studied in LS and exhibits distinct characteristics, including multiple copies per cell and higher mutation frequency. Changes in mtDNA copy number, sequence, mitochondrial displacement loop and mitochondrial MSI (mtMSI) have all been reported in CRC. Still, there is no consensus in the literature about their role in the diagnosis and prognosis [120]. Only a few authors have reported liquid biopsies. Thyagarajan et al. [94] noted that altered mtDNA copy number in peripheral blood is more likely to be a marker of early CRC than CRC risk or oxidative stress, which might make it all the more useful for the screening of LS patients.

## 5. Cell-Free RNA

Analysis of RNA molecules is a relatively novel approach in cancer diagnostics, aiming to reveal dysregulations of gene expression and alternative splicing. With progressive improvements in the field of molecular characterisation, there is considerable potential to identify RNA markers that may support clinical decision making in CRC and ultimately in families with a positive history of LS.

### 5.1. mRNA

MLH1 mRNA in blood samples may serve as a promising biomarker for detecting and distinguishing LS patients from healthy individuals, with an estimated sensitivity and specificity of up to 82% and 87%, respectively [95]. Sequence variants identified in the genetic screening of MMR genes have the potential to directly affect gene expression by altering mRNA splicing, transcription levels, polyadenylation and/or RNA stability. Approximately 30% of reported MMR variants disrupt normal RNA splicing [121]. A large cohort study on nearly 370 patients with LS showed that 40% of patients are carriers of an MLH1 mutation, with the most frequent type of alteration being a change affecting a splice site [122].

### 5.2. Non-Coding RNA

It is becoming increasingly clear that non-coding RNAs (ncRNAs) obtained from liquid biopsies will play a considerable role as diagnostic and prognostic biomarkers in various cancer types in the near future [123]. Their most well-known class, miRNAs, are ≈22 nucleotides long and regulate target mRNAs at the transcriptional and post-transcriptional level [124]. Tumour-derived cell-free miRNAs may be relatively easily separated, and their expression profiles are relevant as markers of early diagnosis and relapse [125]. In CRC, serum miR-1247-5p, miR-1293, miR-548at-5p, miR-107 and miR-139-3p were shown to be differentially expressed between benign adenomas and precancerous polyps or colon cancer, making them ideal candidates for liquid biopsy-based screening in LS patients [99].

In 2016, Zhou et al. determined miR-137, miR-520e and miR-590-3p to be differentially expressed in LS [126]. The year before, Kaur et al. searched for novel epigenetically silenced tumour suppressor miRNAs and found that the hypermethylation of miR-345 and miR-132 was associated with MMR-D CRC, while the hypermethylation of miR-132 allowed differentiating between sporadic MMR-D CRC from tumours that develop on the background of LS. They also reported that the methylation of several miRNAs (most notably, miR-129-2) may serve as a marker of progression in early EC in LS [127]. It has been suggested before that methylation-based markers might prove useful in non-invasive, early detection of malignancies, but the studies need to be repeated with peripheral blood first [128]. miRNA profiling of CRC is not only suitable to detect early disease, but it allows LS-associated tumours to be distinguished from sporadic MSI-H tumours based on differences in the expression of certain miRNAs such as miR-622, miR-1238 and miR-192 [129]. Low expression of some tumour suppressor miRNAs (such as miR-21, miR-34a and miR-126) detectable from serum samples may not be relevant for prognosis but may be used as early detection markers of CRC [100]. Another tumour suppressor miRNA, mir-133b, is a confirmed biomarker in peripheral blood for a number of LS-associated tumours such as CRC [98], gastric [96] and bladder cancer [97], and it has been suggested in ovarian cancer [130] although its usefulness in the latter is not yet confirmed from liquid biopsy to our knowledge.

lncRNAs are another class of non-coding RNAs involved in regulating gene expression at multiple levels [131]. Recently, they were reported to show some promise as markers of early-stage CRC [132]. lncRNAs contribute to carcinogenesis and tumour progression by affecting the WNT/beta-catenin, PI3K/Akt, EGFR, NOTCH, mTOR and TP53 signalling pathways. Moreover, they may influence chemoresistance by acting as miRNA sponges. Some lncRNAs (CCAT1, CCAT2, BLACAT1, CRNDE, NEAT1, UCA1) have been suggested as biomarker candidates for liquid biopsy-based diagnostic CRC tests [133]. lncRNA BCAR4 may serve as a robust CRC marker from peripheral blood in combination with two mRNAs, KRTAP5-4 and MAGEA3 [107]. The number of currently known lncRNA biomarkers is not in proportion with the magnitude of the suspected role of lncRNAs in the disease; further research is likely to reveal more candidates.

## 6. Exosomes

**Figure 2:**
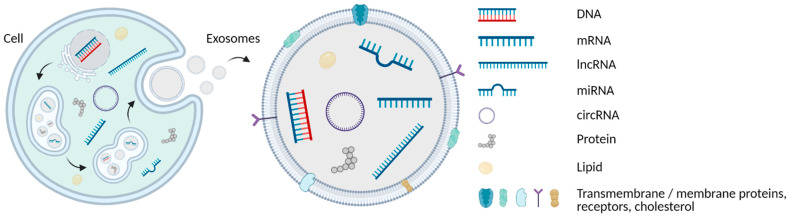
Formation of exosomes. Schematic illustration of the genesis and content of multivesicular bodies (MVB) formed from the endoplasmic reticulum (ER) and producing exosomes. They may be loaded with various cargo, such as cfDNA of nuclear and mitochondrial origin, multiple types of cfRNA, proteins and lipids, found both on the inside and on the surface of exosomes. Their phospholipid bilayer contains additional components: proteins, membrane receptors, etc. (Created with BioRender.com).

Exosomes are defined as extracellular lipid bilayer vesicles with a diameter of 30 to 100 nm secreted from several cell types. They carry DNA, RNA, lipids and proteins both in their lumen and bound on their surface and play essential roles in cell-to-cell communication (Figure 2) [80,134]. Exosomes are more widely investigated in cancer compared to other types of EMVs such as microvesicles and apoptotic bodies [135]. Tumour-derived exosomes affect the immune response, regulate chemoresistance, and were recently suggested as biomarkers of early CRC [136]. Hon et al. (2019) demonstrated the transfer of drug resistance to sensitive cells via exosomes. They found 105 upregulated and 34 downregulated circRNAs in a FOLFOX-resistant HCT116-R colon cancer cell line, concluding that hsa_circ_0000338 isolated from exosomes may serve as an early predictor of chemoresistance [134].

The purification of exosomes from extracellular fluid still poses a challenge as no method is currently accepted as a gold standard [136]. Techniques tried so far include ultracentrifugation, size-exclusion chromatography [137], precipitation-based and column-based isolation kits such as the commercially available ExoQuick and Exospin [138], immunoplate- and immunobead-based affinity isolation [139], and Tim4 purification, which involves the binding of Tim4 to phosphatidylserine exposed on the surface of exosomes and releasing it by adding Ca chelators [140]. Exochips and electrophoretic sorting platforms using various approaches—such as the direct current-insulator-based dielectrophoretic (DC-iDEP) method—seem like promising innovations, offering the advantage of high throughput, speed and sensitivity with minimal sample handling [141]. Nowadays, it is strongly believed that exosomes play an important role in the metastatic process [142]. Numerous studies have also suggested a possible role of exosomes in the early diagnosis and prognosis of LS-related ovarian cancer [143,144,145].

On a side note, exosomes may have a role in CRC therapy as well as diagnostics. Dendritic cells are known to take up tumour antigens contained in exosomes and present them to tumour-specific T-lymphocytes. Animal studies on antitumour responses provoked by vaccination with such exosomes look promising [146].

## 7. Conclusions

In this review, we attempted to share our view on how the diagnostics of LS may be improved and how tumour screening during the follow-up of LS patients may be revolutionised in the near future by the use of nucleic acid biomarkers obtained from liquid biopsies. Progress has been made towards the first step on this path: identifying potential biomarkers. The next step involves confirming and standardising these markers and developing cost-efficient testing methods. In recent years, the increasing accuracy and constantly decreasing costs of high-throughput sequencing contributed to a steady spread of NGS technology. Genetic testing of newly diagnosed CRC patients (even those with asymptomatic relatives) would benefit the patient and possible at-risk family members. At the same time, innovations in computational algorithms allow extensive screening for numerous biomarkers in tens to hundreds of samples simultaneously, with sensitivity comparable to conventionally used methods. While the detection of non-malignant tumours is still out of reach, diagnosing early-stage cancer will vastly improve prognosis in LS. Notably, amid all constructive debate on the utility of liquid-based biopsy samples versus traditionally used tissue specimens, there is space for improvement in laboratory and bioinformatics infrastructure to accommodate new knowledge and new methods.

There is no need to explain how heavy a burden a genetic cancer predisposition syndrome may be. However, there are definite signs that allow us to be optimistic about the future of minimally invasive diagnostics. The question is not whether the need for regular colonoscopy may eventually be eliminated so that an LS patient may just walk into their physician’s office for a quick blood test. The only question seems to be when we will get there.

## Figures and Tables

**Table 1:** Tissue-based methods for MSI screening to distinguish sporadic tumours from lynch syndrome.

Method	Advantages	Limitations
IHC	Workflow takes up to 4–6 h	Analysis of MMR proteins separately
	Easy to perform	Needs a pathologist with experience in MMR IHC interpretation
	Performable in samples with <20% neoplastic cells Able to identify defective MMR genes for downstream analysis	Equivocal test results due to the heterogeneous expression of MMR proteins False-positive results (artificial loss of expression) due to pre-analytic issues or lack of technical calibration Rare false-negative results if there is no apparent loss of expression due to missense mutations in the MMR genes with intact immunoreactivity in approximately 10% of all cases Not reliable in small biopsy specimens Sensitivity depends on antibody panel
MSI-PCR	Allows simultaneous detection of multiple targets	No indication about MMR genes
	Highly reproducible Workflow takes less than 5 h	Requires samples with at least 20% neoplastic cells Rare false-positive results due to microsatellite polymorphisms Informative only for a few tumour types Limited number of markers

**Table 2:** Confirmed circulating nucleic acid biomarkers obtained from liquid biopsy, applicable in lynch syndrome.

Class	Target	Application in LS	Method	References
cfDNA (nuclear origin)	Alu	MSI status assessment	Inter-Alu-PCR, NGS	[88]
	whole exome	MSI status assessment	MSIsensor-ct	[89]
	BAT26, ACVR2A, DEFB105A/B	MSI status assessment	ddPCR	[90]
	*MLH1* promoter	methylation status assessment	ddPCR	[91]
	BAT25, BAT26, MONO27, NR21, NR24	MSI status assessment	ddPCR, NGS	[92]
	*TERT* promoter, *FGFR3*	UC screening	NGS	[93]
cf-mtDNA	*ND1* copy number	CRC screening	qPCR	[94]
cf-mRNA	MLH1	LS diagnosis	qRT-PCR	[95]
cf-miRNA	miR-133b	Screening for various LS-associated malignancies	qRT-PCR	[96,97,98]
	miR-1247-5p, miR-1293, miR-548at-5p, miR-107, miR-139-3p	CRC screening	microarray, qRT-PCR	[99]
	miR-21, miR-34a, miR-126	CRC screening	qRT-PCR	[100]
cf-lncRNA	CCAT1, CCAT2, BLACAT1, CRNDE, NEAT1, UCA1	CRC screening	qRT-PCR	[101,102,103,104,105,106]
	BCAR4 (combined with mRNA markers)	CRC screening	qRT-PCR	[107]
